# Identification of platelet function-related genes in STEMI patients

**DOI:** 10.3389/fgene.2025.1651794

**Published:** 2025-09-22

**Authors:** Jingjing Zhu, Shuangya Yang, Qing Guo, Yifan Yang, Bei Shi

**Affiliations:** Department of Cardiology, Affiliated Hospital of Zunyi Medical University, Zunyi, China

**Keywords:** STEMI, key gene, platelet-related genes, diagnosis, CVD

## Abstract

**Background:**

ST-elevation myocardial infarction (STEMI) is characterized by extensive myocardial necrosis due to acute and severe ischemia, with platelets playing a key role in its pathogenesis. This study aimed to identify platelet function-related biomarkers in STEMI patients.

**Methods:**

The GSE59867 dataset, including STEMI patients and controls, was analyzed to identify differentially expressed genes (DEGs) using the limma R package. Platelet function-related DEGs (DEPRGs) were obtained by intersecting DEGs with platelet-related genes. Gene ontology (GO) and kyoto encyclopedia of genes and genomes (KEGG) enrichment analyses were performed, and a protein-protein interaction (PPI) network was constructed using STRING, followed by identification of hub genes via Cytohubba. The diagnostic value of these genes was evaluated through receiver operating characteristic (ROC) analysis, and further expression validation along with ROC validation was conducted in the GSE123342 dataset. Additionally, gene expression was validated by quantitative real-time polymerase chain reaction (RT-qPCR) in peripheral blood from STEMI patients and cardiac tissue from STEMI mouse models.

**Results:**

A total of 245 DEPRGs were identified; enrichment analyses revealed their primary involvement in hemostasis, coagulation, and platelet activation (adjusted *P* < 0.05). The PPI network screening identified 11 hub genes, among which GRB2, MAPK1 (ERK2), MAPK3 (ERK1), PIK3CA, AKT1, and PIK3R1 demonstrated strong diagnostic performance (AUC > 0.7). ROC analysis yielded the following AUC values (95% CI): GRB2 0.759 (0.678–0.835), MAPK1 0.736 (0.650–0.810), MAPK3 0.824 (0.752–0.885), PIK3CA 0.806 (0.735–0.868), AKT1 0.724 (0.633–0.807), and PIK3R1 0.809 (0.732–0.879); all P-values were <0.05 after adjustment for multiple comparisons. Further validation in an independent dataset confirmed that MAPK3 (*P* = 1.6 × 10^−5^) and GRB2 (*P* = 8.2 × 10^−7^) exhibited consistent expression trends with the training set, with ROC analysis showing AUC values greater than 0.7 for both genes (MAPK3: AUC = 0.808 [95% CI: 0.709–0.899]; GRB2: 0.759 AUC = [95% CI: 0.765–0.929]). Thus, MAPK3 and GRB2 were identified as key genes. qPCR validation in peripheral blood from STEMI patients (n = 30) and cardiac tissue from a mouse myocardial infarction model further confirmed the differential expression of GRB2 and MAPK3 (*P* < 0.05).

**Conclusion:**

This study identified two platelet function-related genes, MAPK3 and GRB2, as potential biomarkers for STEMI, demonstrating high clinical relevance and diagnostic value.

## Graphical Abstract

**Figure:**
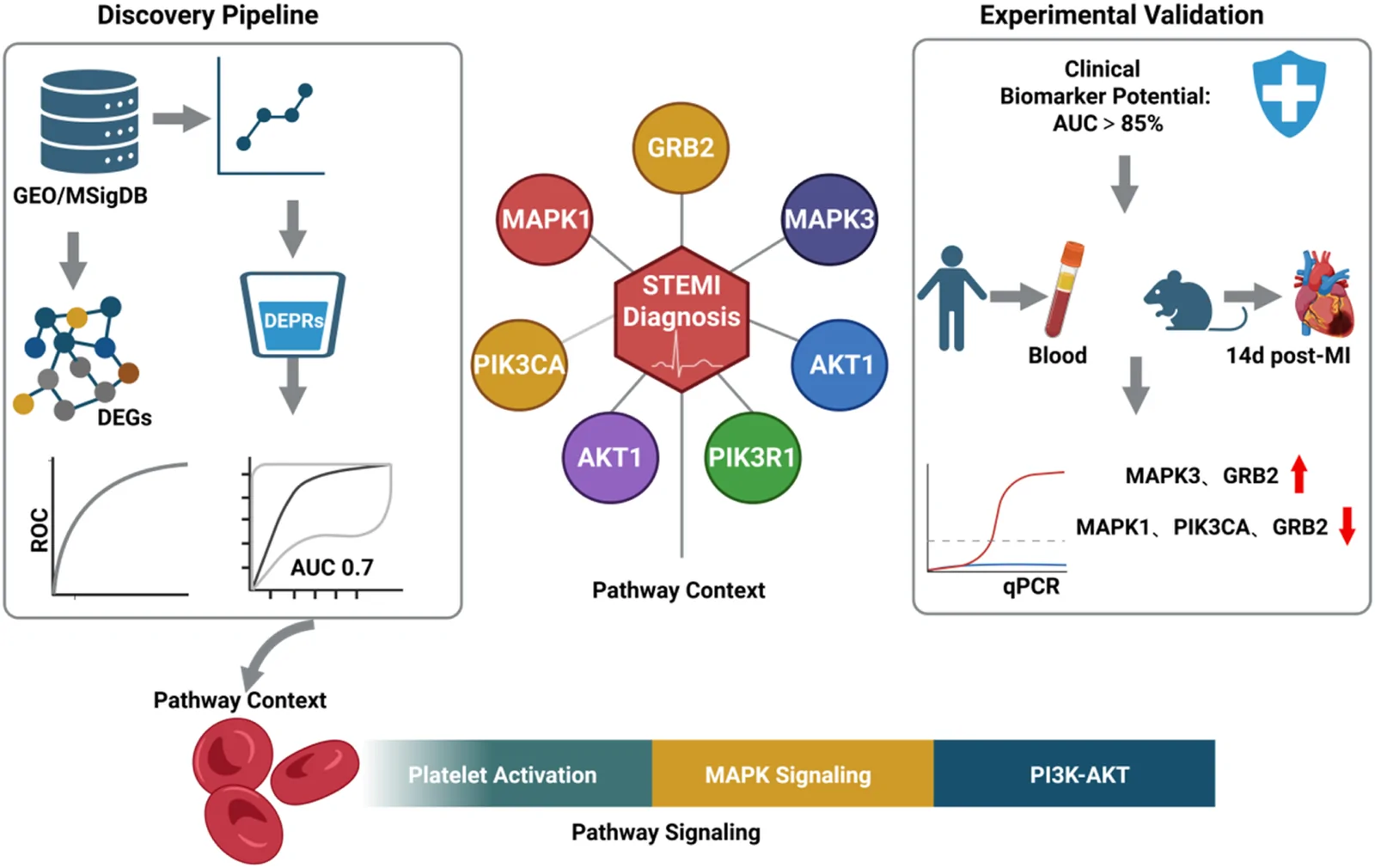
Created with BioRender.com.

## Introduction

Myocardial infarction (MI) remains the foremost global cause of mortality and disability, responsible for >16% of worldwide deaths. While advances in interventional therapies and secondary prevention have reduced MI mortality by 30%–50% in high-income nations over the past decade (GBD, 2021 ASEAN Cardiovascular Diseases Collaborators, 2025), developing countries face a paradoxical rise in disease burden.Acute ST-segment elevation MI (STEMI) – a critical manifestation of acute coronary syndrome (ACS) – involves ischemic cardiomyocyte necrosis due to abrupt coronary flow cessation. Its core pathology stems from atherosclerotic plaque rupture/erosion triggering thrombosis and coronary occlusion, thereby creating myocardial oxygen supply-demand imbalance (Bergmark et al., 2022; Byrne et al., 2023). Current management hinges on:Early reperfusion (thrombolysis/primary PCI), Pharmacotherapy (antiplatelets, statins, β-blockers) (Kumar and Golwala, 2022; Khattak et al., 2024). Although these strategies reduce acute mortality, *persistent challenges* in rapid diagnosis precision and individualized therapeutic optimization demand urgent resolution.

In the pathogenesis of acute myocardial infarction (MI), platelets play a pivotal role in coronary artery thrombosis and disease progression (Işık and Soner, 2022),However, there remains a lack of well-established biomarkers for the assessment of platelet dysfunction in clinical practice. Genomic and molecular studies have identified several platelet-associated genes, such as P2RY12 and integrin αIIbβ3 (GPIIb/IIIa), which are significantly associated with susceptibility to MI and thrombotic risk (Keramati et al., 2021; Stimpfle et al., 2018; De Vries et al., 2015); Additionally, epigenetic regulators, including platelet-derived miR-223, miR-126, and histone deacetylase 6 (HDAC6), contribute to platelet activation and thrombus formation, thereby participating in the pathological process of thrombosis (Corti et al., 2003; Gawaz, 2006; Chen et al., 2022; Sun et al., 2019). Although these studies have provided valuable insights into the mechanisms underlying infarction-related thrombosis, current diagnostic biomarkers still lack sufficient sensitivity and specificity. Moreover, the clinical translation of potential therapeutic targets remains constrained. Thus, the identification and development of high-performance biomarkers are essential for optimizing the clinical management of ST-segment elevation myocardial infarction (STEMI).

This study employs bioinformatics and experimental validation to identify and characterize platelet function-related gene signatures in STEMI patients, aiming to assess their mechanistic involvement in acute coronary thrombosis and evaluate their diagnostic potential.

### Data source

The RNA expression datasets GSE59867 and GSE123342 were downloaded from the Gene Expression Omnibus (GEO; https://www.ncbi.nlm.nih.gov/geo/). In this study, the GSE59867 dataset was based on the platform GPL6244 [HuGene-1_0-st] Affymetrix Human Gene 1.0 ST Array [transcript (gene) version], and was comprised of 111 MI periperal blood mononuclear cells (PBMCs) samples (on the first day of MI-admission) and 46 control periperal blood mononuclear cells (PBMCs) samples. The dataset GSE123342 was utilized as the validation set, comprising 65 PBMC samples from MI patients and 22 PBMC samples from control subjects. We obtained 480 platelet-related genes from the MSigDB database.

### Identification of DEGs

To identify differentially expressed genes (DEGs) between MI and control groups, the Limma R package (version 3.58.1) (Ritchie et al., 2015) was employed to evaluate gene expression differences between MI and control samples in the GSE59867 dataset (Maciejak et al., 2015). Genes meeting the cutoff criteria of adjusted *P*-value <0.05, were considered as DEGs. The “ggplot2 (3.5.2)” (Ito and Murphy, 2013) and “pheatmap (1.0.12)” R packages (10.32614/CRAN.package.pheatmap) were used to visualize the DEGs between the MI and control samples. The top 10 upregulated and 10 downregulated genes, ranked by log fold-change (logFC), were selected for visualization.

### KEGG and GO enrichment analysis

To obtain platelet-related DEGs, a cross-analysis between DEGs and platelet-associated genes was performed using the “VennDiagram (0.1.10)” R package (10.32614/cran.package.ggvenn), and the resulting intersections were defined as DEPRGs. Functional annotation of DEPRGs was presented with the R package “clusterProfiler (4.8.3)” (Yu et al., 2012), containing GO and KEGG pathway analyses. GO terms were comprised of the biological process (BP), cellular component (CC), and molecular function (MF) (The Gene Ontology Conso rtium, 2017). KEGG pathway analysis was performed to obtain the associated enrichment pathways. Adjust *P*-value < 0.05 was considered statistically significant.

### Construction of protein-protein interaction (PPI) network

To investigate the protein-level interaction relationships among candidate genes, the STRING database as employed to construct a protein-protein interaction (PPI) network for the DEPRGs (Szklarczyk et al., 2015). The CytoHubba plugin in Cytoscape was utilized to identify hub genes within the PPI network, with the top 10 nodes ranked by Degree selected as core genes. Additionally, the Corrplot R package was applied to analyze the correlations among these hub genes.

### Expression analysis and the ROC curve analysis

The expression levels of hub genes were validated using the Wilcoxon rank-sum test, and their differential expression between MI and control samples was visualized through boxplots generated with the “ggplot2” R package. ROC analysis was performed to evaluate whether hub genes could differentiate MI samples from control samples using the “pROC” R package (1.18.5) (Robin et al., 2011). Hub genes with an area under the ROC curve (AUC) > 0.7 were identified as diagnostic genes for myocardial infarction. Genes exhibiting AUC >0.7 and significant expression differences in the training set were further validated in the GSE123342 dataset. Genes that showed significant differences between the training set and the validation set, had consistent expression trends, and an AUC >0.7 were identified as Key genes.

### GSEA analysis and correlation analysis

To further explore the signaling pathways and underlying biological mechanisms in which the key genes were involved. We used the R package “clusterprofiler (4.8.3)” to conduct GSEA on diagnostic biomarkers (Bechhofer et al., 1986). The reference gene sets used in this analysis were downloaded from the Molecular Signatures Database (MSigDB), primarily utilizing KEGG gene sets as the background gene sets. NES>1 and Adjust *P*-value < 0.05 was considered statistically significant. The top 5 significantly enriched results were presented based on their significance ranking. To analyze the correlation between key genes, Spearman correlation analysis was conducted on all samples in the training set using the corr.test function from the R package “psych” (with thresholds of |cor| > 0.3 and *p* < 0.05). The results were visualized using the R package “ggplot2”.

### Prediction of networks regulated by miRNAs and TFs

To investigate the mutual regulatory network of key genes, the upstream transcription factors (TF) and the miRNAs were predicted using the miRNet database (Fan and Xia, 2018). We choose the miRNA of degree >2 for visualization. Subsequently, the TF-target genes and miRNA-target genes regulatory networks were visualized by Cytoscape software.

### Inclusion and exclusion criteria for human peripheral blood samples

STEMI patients were eligible for inclusion if they met all of the following: (i) age between 18 and 80 years; (ii) diagnosis of acute ST-segment elevation myocardial infarction according to the 2023 ESC Guidelines for the management of acute coronary syndromes (Byrne et al., 2024), defined by typical chest pain lasting ≥20 min, new ST-segment elevation in ≥2 contiguous leads on 12-lead ECG, and elevated cardiac troponin T/I; (iii) presentation within 12 h of symptom onset; (iv) planned primary percutaneous coronary intervention (PCI). Stable coronary artery disease (SCAD) controls were included if they had angiographically confirmed coronary artery stenosis of 50%–70% without acute ischemic symptoms, and no evidence of recent myocardial infarction (within 6 months). For both groups included: history of previous MI or coronary revascularization; active infection or inflammatory disease; hematologic disorders affecting platelet count or function; malignancy; severe hepatic or renal dysfunction (estimated GFR <30 mL/min/1.73 m^2^); autoimmune diseases; recent major surgery or trauma (within 3 months); current use of anticoagulant or antiplatelet agents other than aspirin or clopidogrel within the preceding 14 days; and refusal to provide informed consent.

### Peripheral blood mononuclear cell isolation

Peripheral blood samples (2 mL each) were collected from 30 consecutive STEMI patients at emergency admission prior to emergency percutaneous coronary intervention (PCI), along with 30 age- and sex-matched stable coronary artery disease (SCAD) controls (Coronary angiography revealed 50%–70% stenosis in the target coronary artery). The baseline characteristics of the patients are presented in Table 1. For lymphocyte isolation, whole blood samples were carefully layered onto Ficoll-Paque density gradient medium (LTS1077; TBD Science, China) at a 1:1 volume ratio in 50 mL conical tubes. After gentle mixing, samples were centrifuged at 2,000 × g for 20 min at room temperature. The distinct lymphocyte layer was subsequently aspirated and transferred to a fresh centrifuge tube. Cells were washed twice with phosphate-buffered saline (PBS) through sequential centrifugation (1,000 × g, 10 min, 4 °C) to remove residual plasma components. All participants provided written informed consent. The study protocol was approved by the Ethics Committee of The Affiliated Hospital of Zunyi Medical University (Approval No. KLL-2021-323) and conformed to the Declaration of Helsinki.

**TABLE 1:** Baseline characteristics of SCAD and STEMI Patients.

Characteristic	Overall	SCAD	STEMI	P value^b^
	N = 60^a^	N = 30^a^	N = 30^a^	
Age, years	64.00 (57.00–72.00)	62.00 (57.00–72.00)	65.00 (54.00–72.00)	0.906
HR, bpm	76.50 (68.50–85.00)	77.50 (70.00–85.00)	73.50 (65.00–86.00)	0.756
SBP, mmHg	128.50 (111.50–136.50)	131.50 (117.00–138.00)	127.50 (109.00–135.00)	0.270
DBP,mmHg	75.00 (65.50–83.00)	77.00 (68.00–85.00)	72.00 (62.00–81.00)	0.195
EF, %	54.50 (46.00–60.00)	55.00 (46.00–60.00)	54.50 (46.00–60.00)	0.722
TG, mg/dL	1.46 (1.16–2.32)	1.36 (1.15–2.54)	1.51 (1.17–2.32)	0.965
TC, mg/dL	3.67 (3.13–4.29)	3.52 (3.00–4.09)	3.92 (3.45–4.51)	0.070
HDLC, mg/dL	1.01 (0.92–1.16)	1.02 (0.93–1.17)	1.01 (0.92–1.15)	0.579
LDLC, mg/dL	2.24 (1.74–2.58)	2.06 (1.64–2.54)	2.46 (2.06–2.82)	0.049
Creatinine, mg/dL	84.50 (70.00–93.00)	84.00 (70.00–93.00)	85.00 (70.00–93.00)	0.679
Leukocyte, 10^9^/L	6.82 (5.45–7.95)	6.48 (5.44–8.13)	6.98 (5.45–7.70)	0.779
Redbloodcell, 10^12^/L	4.54 (4.32–4.95)	4.40 (4.16–4.76)	4.76 (4.52–5.10)	0.002
Hemoglobin, g/dL	149.00 (132.00–159.00)	138.50 (127.00–150.00)	155.00 (144.00–183.00)	0.001
Platelet, 10^9^/L	167.50 (141.50–209.00)	168.50 (138.00–209.00)	166.00 (144.00–209.00)	0.751
Neutrophil, 10^9^/L	5.08 (3.80–6.68)	4.85 (3.75–6.28)	5.33 (4.32–6.72)	0.290
NTProBNP, pg/mL	164.00 (77.99–337.50)	152.50 (84.00–257.00)	202.50 (47.34–634.00)	0.375
TnT, ng/mL	20.28 (10.77–152.36)	16.30 (9.54–27.22)	152.36 (13.13–293.40)	<0.001
Sex, n (%)				0.488
Male	50.00 (83.33)	24.00 (80.00)	26.00 (86.67)	
Famale	10.00 (16.67)	6.00 (20.00)	4.00 (13.33)	
Hypertension, n (%)				0.796
No	29.00 (48.33)	46.67 (14.00)	15.00 (50.00)	
Yes	31.00 (51.67)	16.00 (53.33)	15.00 (50.00)	
Diabetes, n (%)				0.573
No	42.00 (70.00)	20.00 (66.67)	22.00 (73.33)	
Yes	18.00 (30.00)	10.00 (33.33)	8.00 (26.67)	
Hyperlipidemia, n (%)				0.796
No	31.00 (51.67)	16.00 (53.33)	15.00 (50.00)	
Yes	29.00 (48.33)	14.00 (46.67)	15.00 (50.00)	
Smoking, n (%)				>0.999
No	22.00 (36.67)	11.00 (36.67)	11.00 (36.67)	
Yes	38.00 (63.33)	19.00 (63.33)	19.00 (63.33)	
ChronicHeartFailure, n (%)				>0.999
No	50.00 (83.33)	25.00 (83.33)	25.00 (83.33)	
Yes	10.00 (16.67)	5.00 (16.67)	5.00 (16.67)	
CKD, n (%)				0.448
No	52.00 (86.67)	25.00 (83.33)	27.00 (90.00)	
Yes	8.00 (13.33)	5.00 (16.67)	3.00 (10.00)	
Aspirin, n (%)				0.542
No	14.00 (23.33)	6.00 (20.00)	8.00 (26.67)	
Yes	46.00 (76.67)	24.00 (80.00)	22.00 (73.33)	
Clopidogrel, n (%)				0.688
No	53.00 (88.33)	26.00 (86.67)	27.00 (90.00)	
Yes	7.00 (11.67)	4.00 (13.33)	3.00 (10.00)	
Statin, n (%)				0.347
No	13.00 (21.67)	5.00 (16.67)	8.00 (26.67)	
Yes	47.00 (78.33)	25.00 (83.33)	22.00 (73.33)	
Beta blocker, n (%)				0.091
No	18.00 (30.00)	6.00 (20.00)	12.00 (40.00)	
Yes	42.00 (70.00)	24.00 (80.00)	18.00 (60.00)	
ACEI, n (%)				0.080
No	16.00 (26.67)	5.00 (16.67)	11.00 (36.67)	
Yes	44.00 (73.33)	25.00 (83.33)	19.00 (63.33)	

^a^ M(IQR) = median (interquartile range); n (%).

^b^ The Mann-Whitney U test was used for continuous variables. The chi-squared test was used for categorical variables.

*HR*, heart rate; *SBP*, systolic blood pressure; *DBP*, diastolic blood pressure; *EF*, ejection fraction; *TG*, triglyceride; *TC*, total cholesterol; *HDL-C*, high-density lipoprotein; *LDL-C*, low-density lipoprotein cholesterol; *RBC*, red blood cell, *NTProBNP* N-terminal Pro-Brain Natriuretic Peptide, *TnT* troponin, *CKD*, chronic kidney disease; *ACEI*, Angiotensin-Converting Enzyme Inhibitor.

### Myocardial infarction model induction

Male C57BL/6 mice (8 weeks old) (Approval No. zyfy-an-2023-0299) were anesthetized via intraperitoneal injection of sodium pentobarbital (50 mg/kg), In brief, the chests were opened through fourth intercostal space and the heart was exposed. The left anterior descending coronary artery (LAD) was ligated with a 6–0 silk suture to create MI. Mice in the sham group received the same experimental procedures but without LAD constriction. After operation, the general condition of mice was monitored daily, and the mice were sacrificed at 2 weeks after MI.

### Quantitative real-time PCR analysis

Cardiac tissues from myocardial infarction (MI) model mice (14 days post-surgery) and peripheral blood mononuclear cells were analyzed by quantitative PCR (qPCR). Total RNA was isolated using TRIzol™ reagent (R0016; Beyotime Biotechnology) after homogenization. RNA concentration was quantified spectrophotometrically, followed by cDNA synthesis with All-in-One First-Strand Synthesis MasterMix (abs60226; Absin Bioscience). The reaction system consisted of: 4 μL of reaction buffer, 1 μL of primers, 1 μL of SweScript RT I enzyme mix, and 0.1 ng to 5 μg of total RNA, with the final volume adjusted to 20 μL using RNase-free water. The qPCR reactions were performed on a QuantStudio 6 Pro system (Applied Biosystems) using Hieff^®^ qPCR SYBR Green Master Mix (Catalog No. 11201ES08; YEASEN). The amplification conditions were as follows: an initial denaturation at 95 °C for 1 min, followed by 40 cycles of denaturation at 95 °C for 20 s, annealing at 55 °C for 20 s, and extension at 72 °C for 30 s. Glyceraldehyde-3-phosphate dehydrogenase (GAPDH) was used as the reference gene, and the relative gene expression levels were calculated using the 2^△△Ct^ method. All primer sequences are detailed in Supplementary Table S1.

### Statistical analysis

All statistical analyses were performed using R software. Differential expression analysis was conducted with the Limma R package (3.58.1); functional annotation was carried out using the R package “clusterProfiler (4.8.3)”; ROC validation was performed with the “pROC” R package (1.18.5); GSEA analysis was implemented via the R package “clusterProfiler (4.8.3)”, and Spearman correlation tests were used for relevant examinations. The significance threshold was set at *p* < 0.05.

## Results

### Identification of DEGs

**FIGURE 1:**
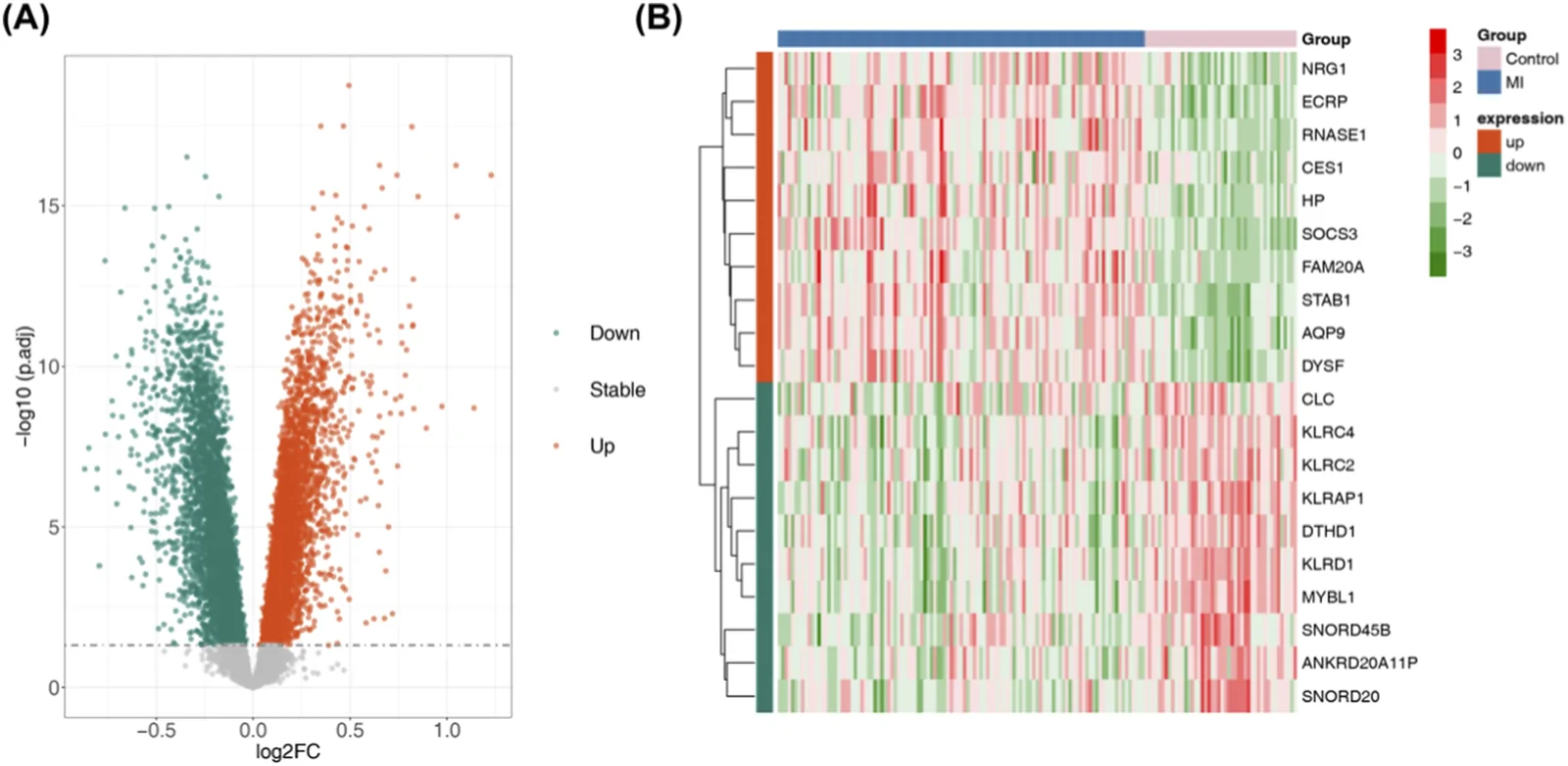
**(A)** Volcano plot of DEGs between MI and control samples in GSE59867. 12,617 DEGs identified (4,457 upregulated and 8,160 downregulated; MI vs. control). **(B)** Heatmap of DEG expression profiles in GSE59867 (MI vs. Control).

In our study, 12,617 DEGs were identified between MI samples and control samples in the GSE59867 dataset. Among them, 4457 were upregulated and 8160 were downregulated (MI vs. control). The volcano plot and heatmap of gene expressions are shown in (Figures 1A,B).

### Identification of DEPRGs and functional enrichment analysis

**FIGURE 2:**
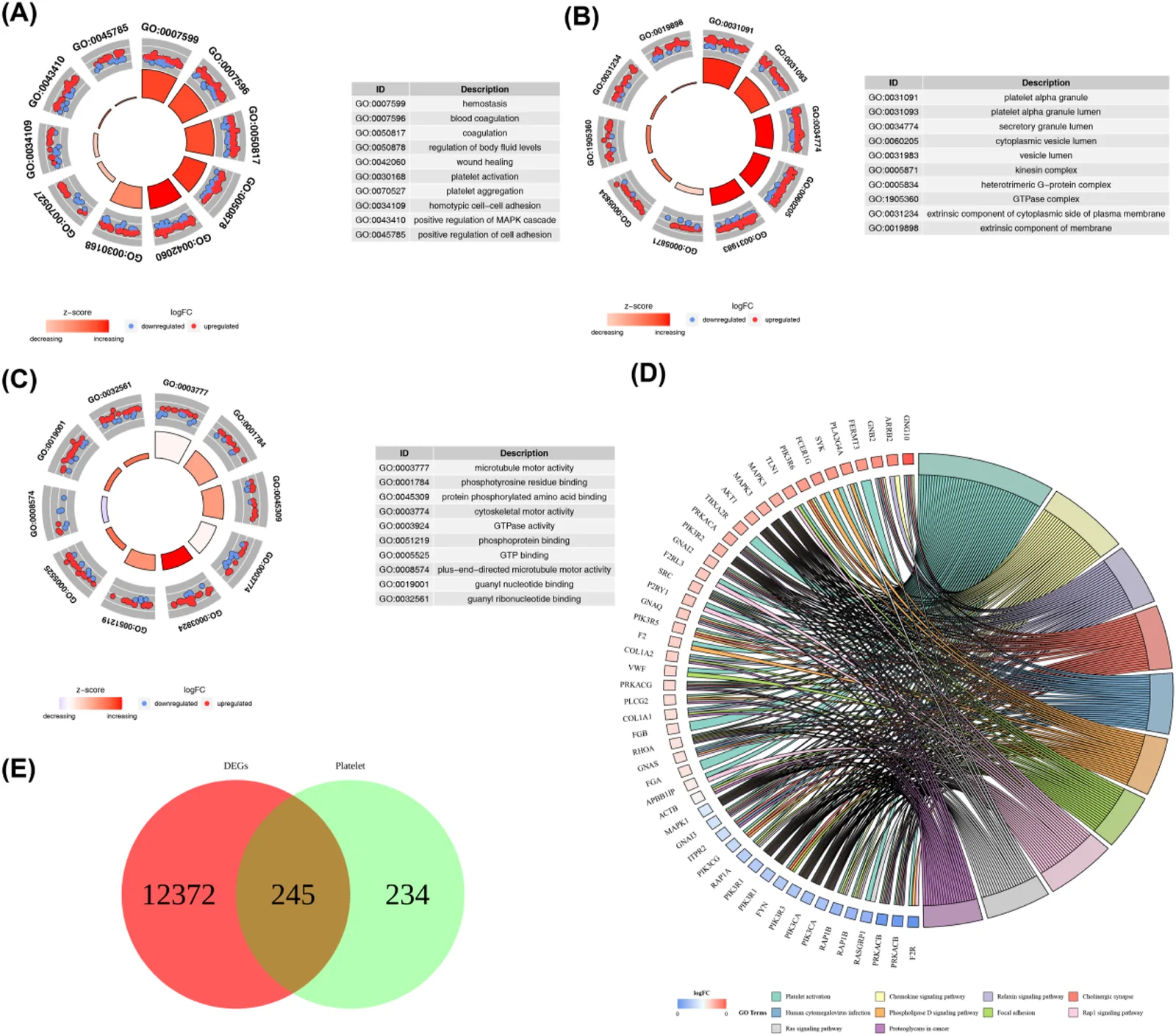
**(A)** GO biological process (BP) enrichment analysis of DEPRGs. Key enriched terms include hemostasis, blood coagulation, coagulation, and regulation of body fluid levels. **(B)** GO cellular component (CC) enrichment analysis of DEPRGs. Significantly enriched components include platelet alpha granule, platelet alpha granule lumen, and secretory granule lumen. **(C)** GO molecular function (MF) enrichment analysis of DEPRGs. Dominant functions include microtubule motor activity, phosphotyrosine residue binding, and protein phosphorylated amino acid binding. **(D)** KEGG pathway enrichment analysis of DEPRGs. Major enriched pathways: platelet activation, chemokine signaling pathway, relaxin signaling pathway, and Ras signaling pathway. **(E)** Venn diagram showing the intersection of differentially expressed genes (DEGs) and platelet-related genes, identifying 245 differentially expressed platelet-related genes (DEPRGs). Total DEGs: 12,617; platelet-related genes: 479; overlapping DEPRGs: 245.

Then, we took the intersection of DEGs and platelet-related genes and identified 245 DEPRGs (Figure 2A). The GO terms were shown in (Figures 2B–D). In BP analysis (Figure 2B), DEPRGs mainly participated in hemostasis, blood coagulation, coagulation, and regulation of body fluid levels. In CC analysis (Figure 2C), DEPRGs significantly participated in platelet alpha granule, platelet alpha granule lumen, and secretory granule lumen. MF analysis showed that DEPRGs significantly participated in microtubule motor activity, phosphotyrosine residue binding, and protein phosphorylated amino acid binding (Figure 2D). The results of KEGG analysis showed that these 245 DEPRGs were mainly enriched in platelet activation, chemokine signaling pathway, and relaxin signaling pathway (Figure 2E).

### Identification of hub genes

**FIGURE 3:**
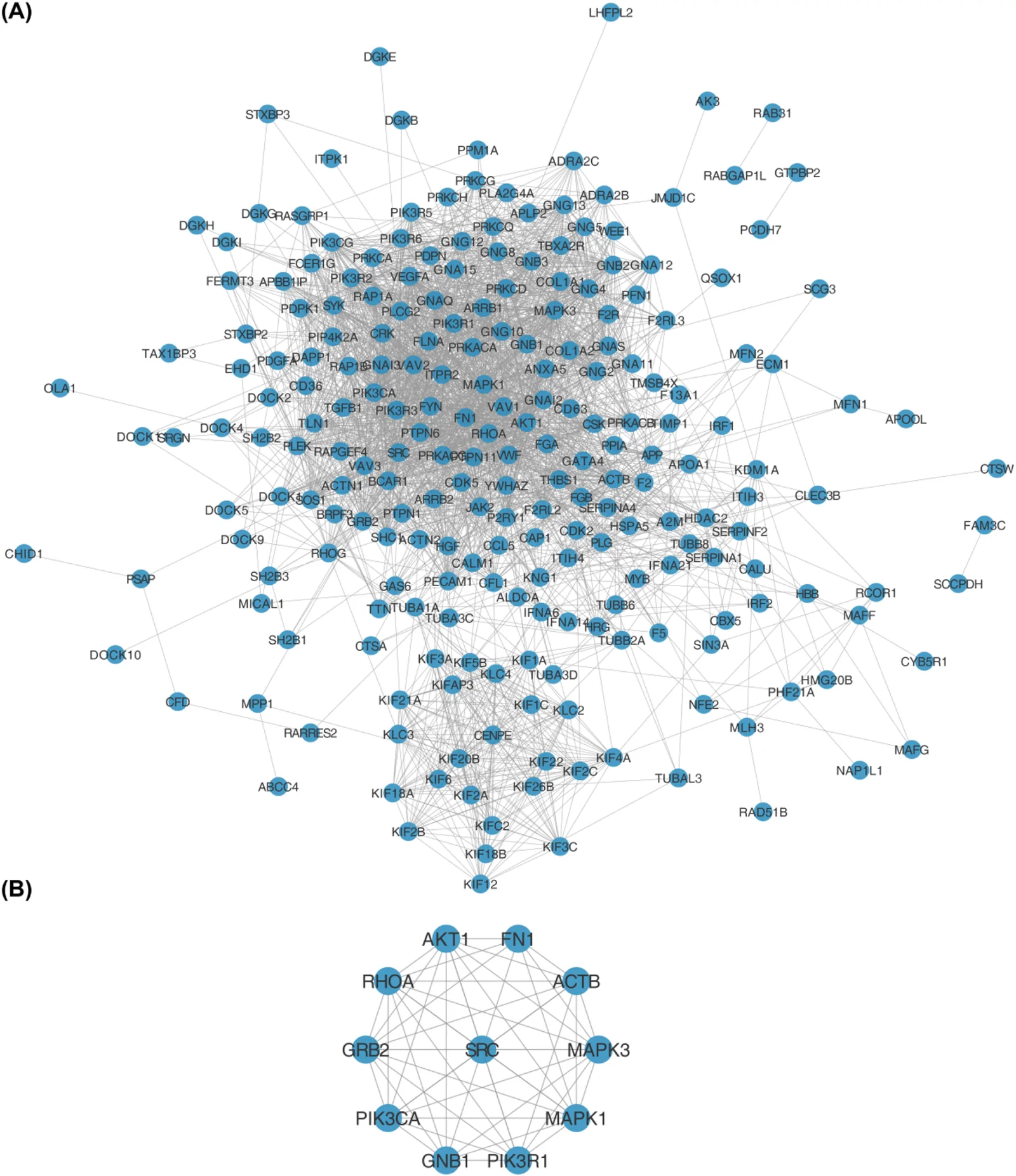
**(A)** Protein-protein interaction (PPI) network of 245 differentially expressed platelet-related genes (DEPRGs) constructed using the STRING database. Nodes represent genes, and edges represent functional interactions. **(B)** Hub genes identified from the PPI network using the CytoHubba plugin in Cytoscape. The 11 hub genes are: AKT1, FN1, ACTB, MAPK3, MAPK1, PIK3R1, GNB1, PIK3CA, GRB2, RHOA, and SRC.

The PPI network was established using the STRING database, and interactions of 245 DEPRGs were displayed in (Figure 3A). 11 hub genes (AKT1, FN1, ACTB, MAPK3, MAPK1, PIK3R1, GNB1, PIK3CA, GRB2, RHOA, and SRC) were identified by CytoHubba plug-in Cytoscape (Figure 3B).

### Determination of key genes

**FIGURE 4:**
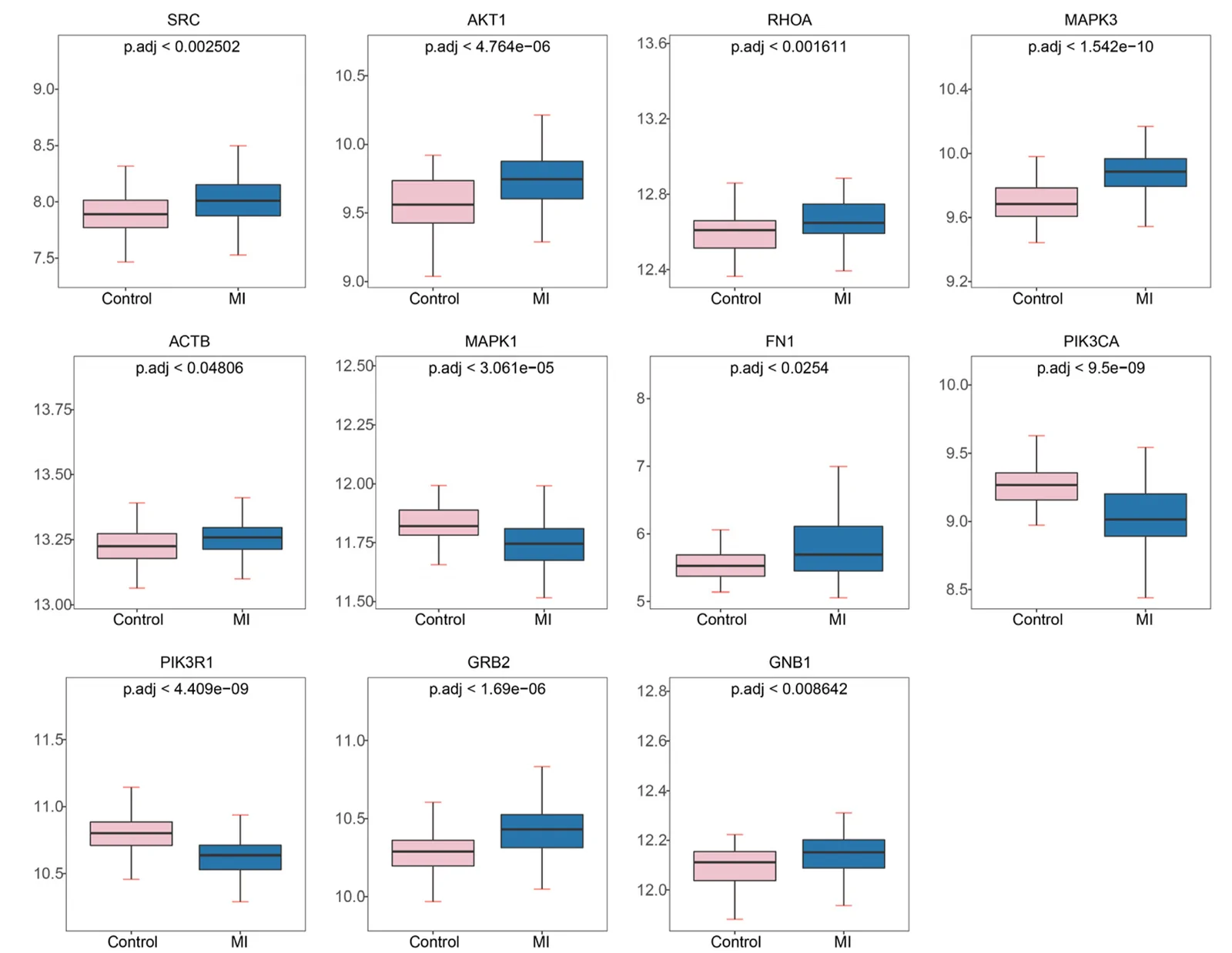
Expression levels of hub genes in myocardial infarction (MI) and control samples from GSE59867. Significantly upregulated genes in MI: SRC, AKT1, RHOA, MAPK3, ACTB, FN1, GNB1, GRB2. Significantly downregulated genes in MI: MAPK1, PIK3CA, PIK3R1. Plots show mean expression values with *p*.adj (adjusted *p*-value) for group comparisons.

**FIGURE 5:**
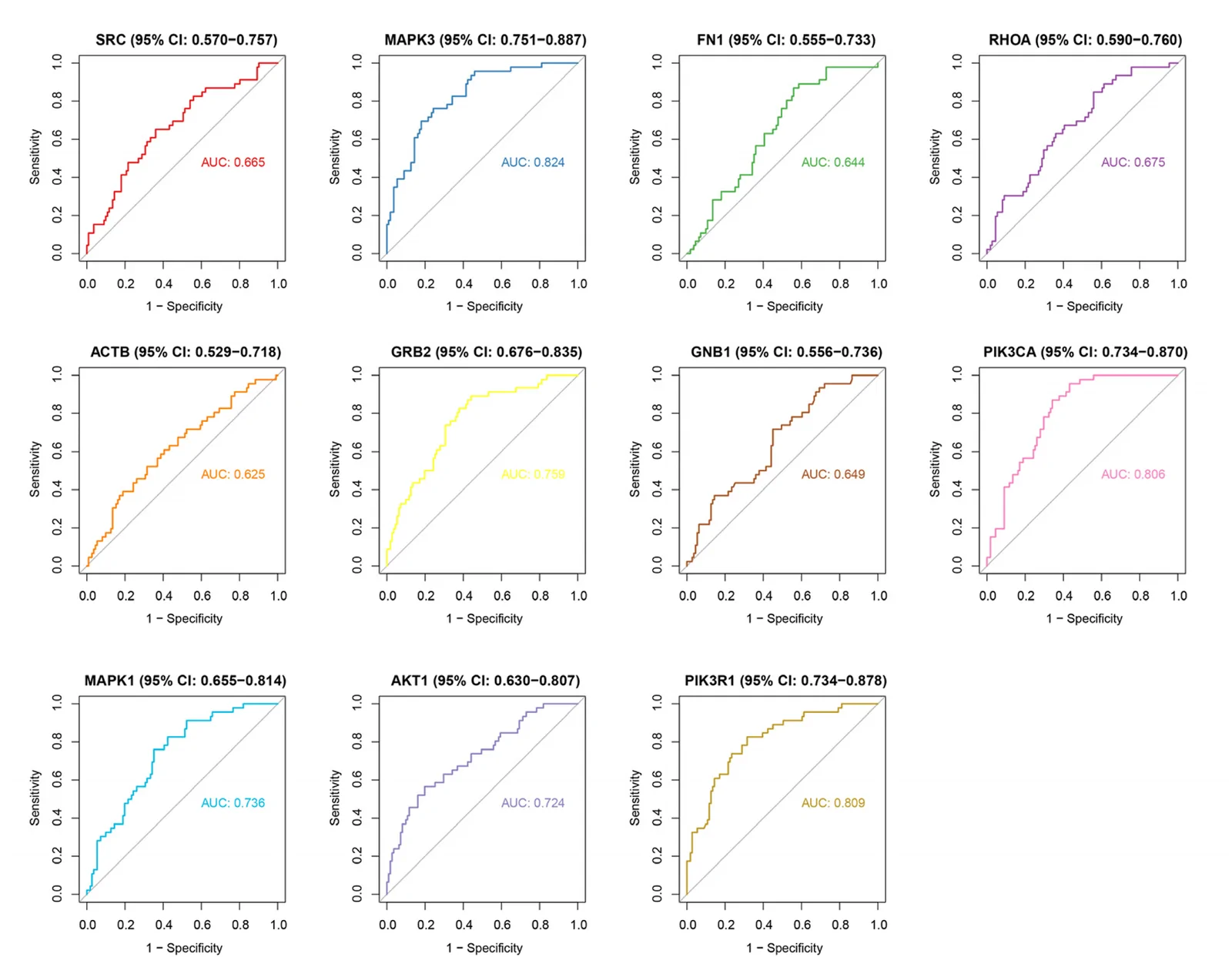
ROC curve analysis of hub genes for MI diagnosis. AUC values: AKT1 (0.724), FN1 (0.644), ACTB (0.625), MAPK3 (0.824), MAPK1 (0.736), PIK3R1 (0.809), GNB1 (0.649), PIK3CA (0.806), GRB2 (0.759), RHOA (0.675), SRC (0.665). Six genes (AKT1, MAPK3, MAPK1, PIK3R1, PIK3CA, GRB2) show high diagnostic accuracy (AUC > 0.7). Curves depict sensitivity vs. specificity for each gene.

**FIGURE 6:**
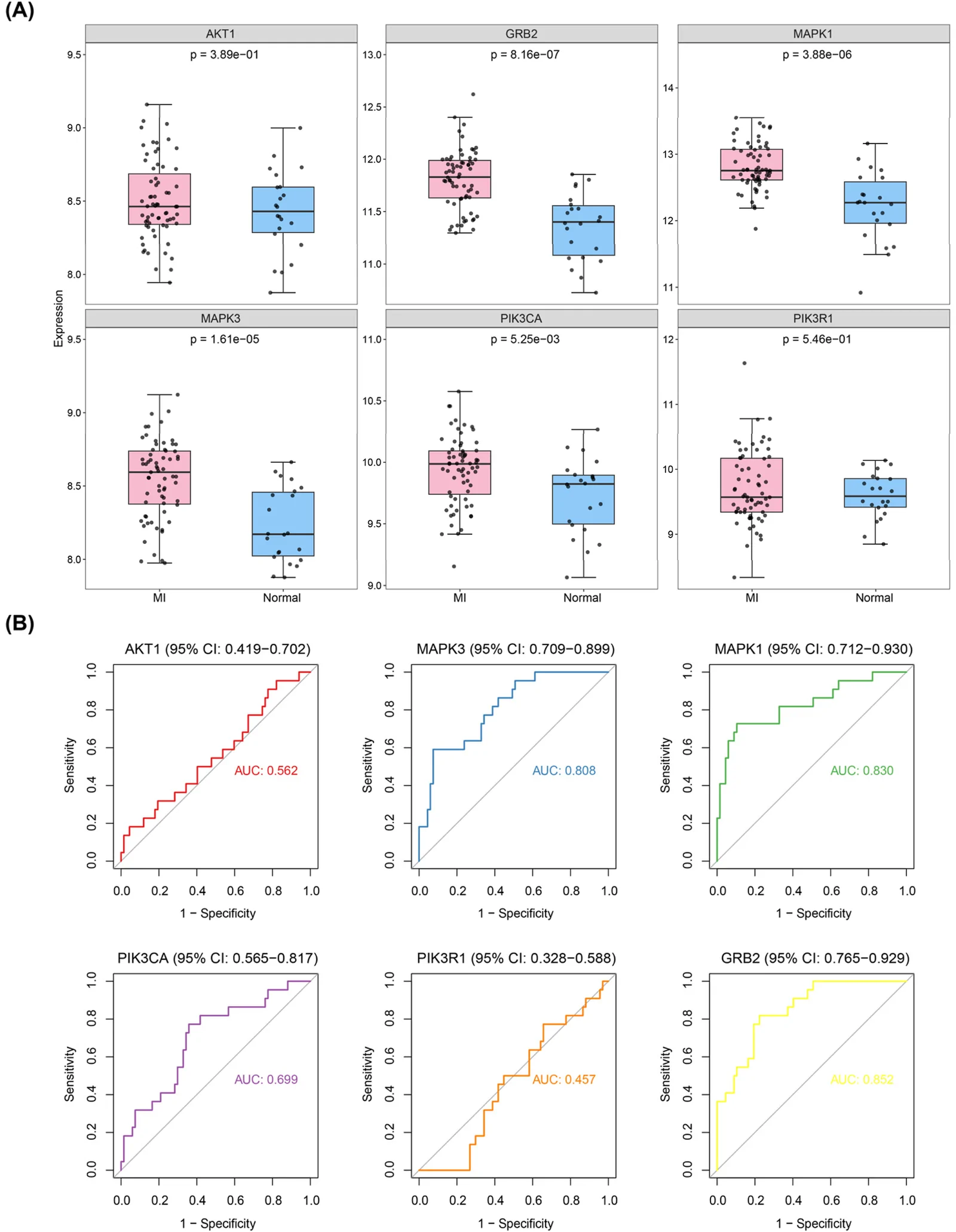
Validation of hub genes in independent datasets. **(A)** Expression validation of candidate hub genes in GSE59867 and GSE123342 datasets. **(B)** ROC curve analysis of the diagnostic performance of candidate hub genes (AKT1, MAPK3, MAPK1, PIK3R1, PIK3CA, and GRB2) in the validation datasets. MAPK3 and GRB2 showed consistent expression trends with the training set and had AUC values > 0.7, indicating their diagnostic potential as key genes.

To investigate the expression patterns of these genes in myocardial infarction (MI) and control samples from the GSE59867 dataset, we found that the expression levels of SRC (*p* < 2.502 × 10^−3^), AKT1 (*p* < 4.764 × 10^−6^), RHOA (*p* < 1.611 × 10^−3^), MAPK3 (*p* < 1.542 × 10^−10^), ACTB (*p* < 4.806 × 10^−2^), FN1 (*p* < 0.0254), GNB1 (*p* < 8.642 × 10^−3^), and GRB2 (*p* < 1.690 × 10^−6^) were significantly higher in MI samples compared to controls, whereas MAPK1 (*p* < 3.061 × 10^−5^), PIK3CA (*p* < 9.500 × 10^−9^), and PIK3R1 (*p* < 4.409 × 10^−9^) showed significantly lower expression in MI samples (Figure 4). As illustrated in Figure 5, we performed ROC curve analysis to evaluate the sensitivity and specificity of these genes in diagnosing myocardial infarction. The AUC values for AKT1, FN1, ACTB, MAPK3, MAPK1, PIK3R1, GNB1, PIK3CA, GRB2, RHOA, and SRC were 0.724 (95% CI: 0.633–0.807), 0.644 (95% CI: 0.557–0.732), 0.625 (95% CI: 0.526–0.722), 0.824 (95% CI: 0.752–0.885), 0.736 (95% CI: 0.650–0.810), 0.809 (95% CI: 0.732–0.879), 0.649 (95% CI: 0.557–0.736), 0.806 (95% CI: 0.735–0.868), 0.759 (95% CI: 0.678–0.835), 0.675 (95% CI: 0.583–0.763), and 0.665 (95% CI: 0.565–0.759), respectively. These results indicated that six genes (AKT1, MAPK3, MAPK1, PIK3R1, PIK3CA, and GRB2) exhibited diagnostic potential. Further validation of these six genes in an independent validation set revealed that MAPK3 (*p* = 1.6 × 10^−5^) and GRB2 (*p* = 8.2 × 10^−7^) maintained consistent expression trends with the training set and showed significant expression differences (Figure 6A). ROC analysis demonstrated that both genes had AUC values greater than 0.7, with MAPK3 having an AUC of 0.808 (95% CI: 0.709–0.899) and GRB2 having an AUC of 0.759 (95% CI: 0.765–0.929). Therefore, MAPK3 and GRB2 were identified as key genes (Figure 6B).

### Correlation analysis between hub genes and GSEA analysis of key genes

**FIGURE 7:**
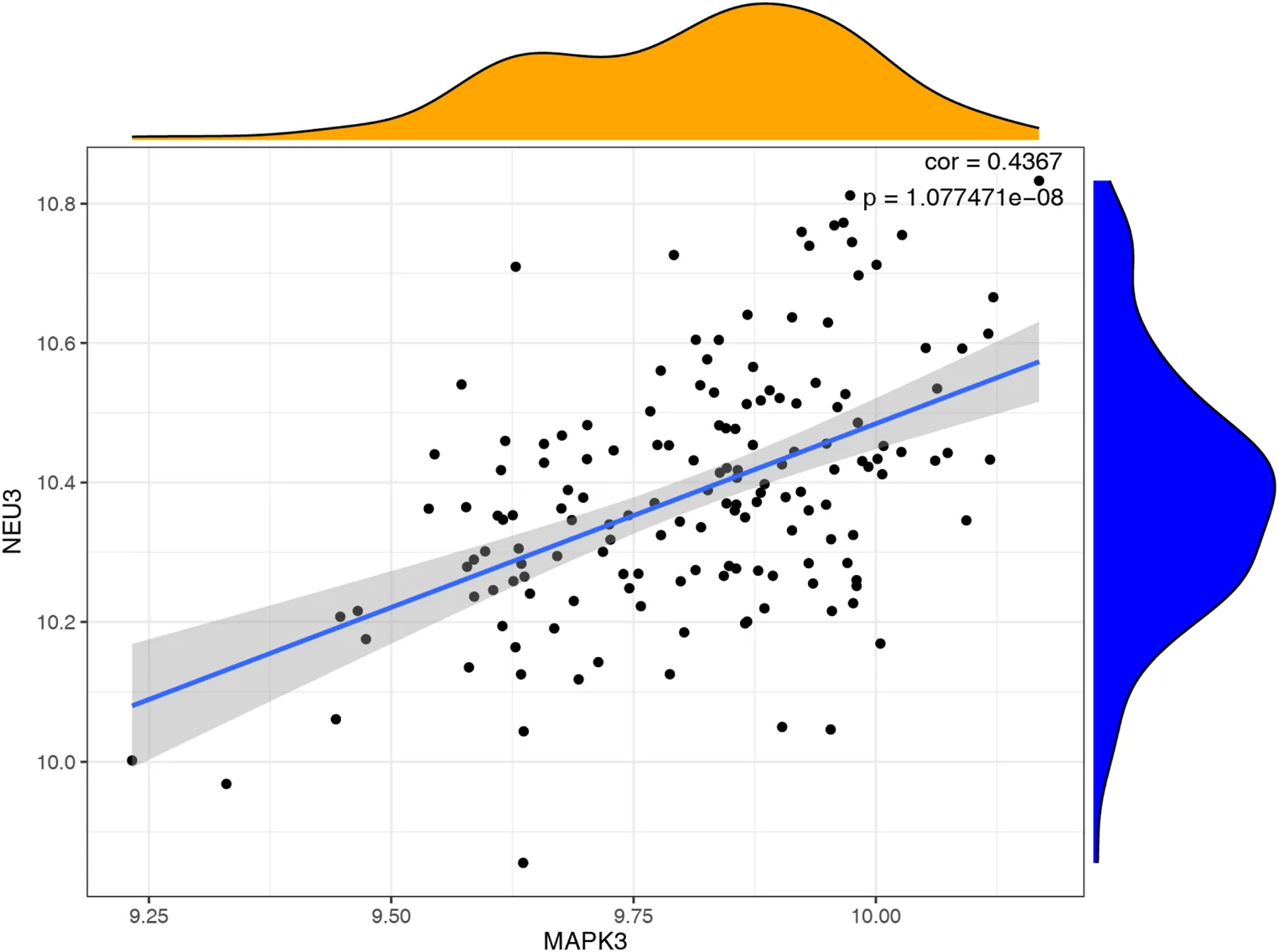
Correlation analysis of key genes using the corrplot package.

**FIGURE 8:**
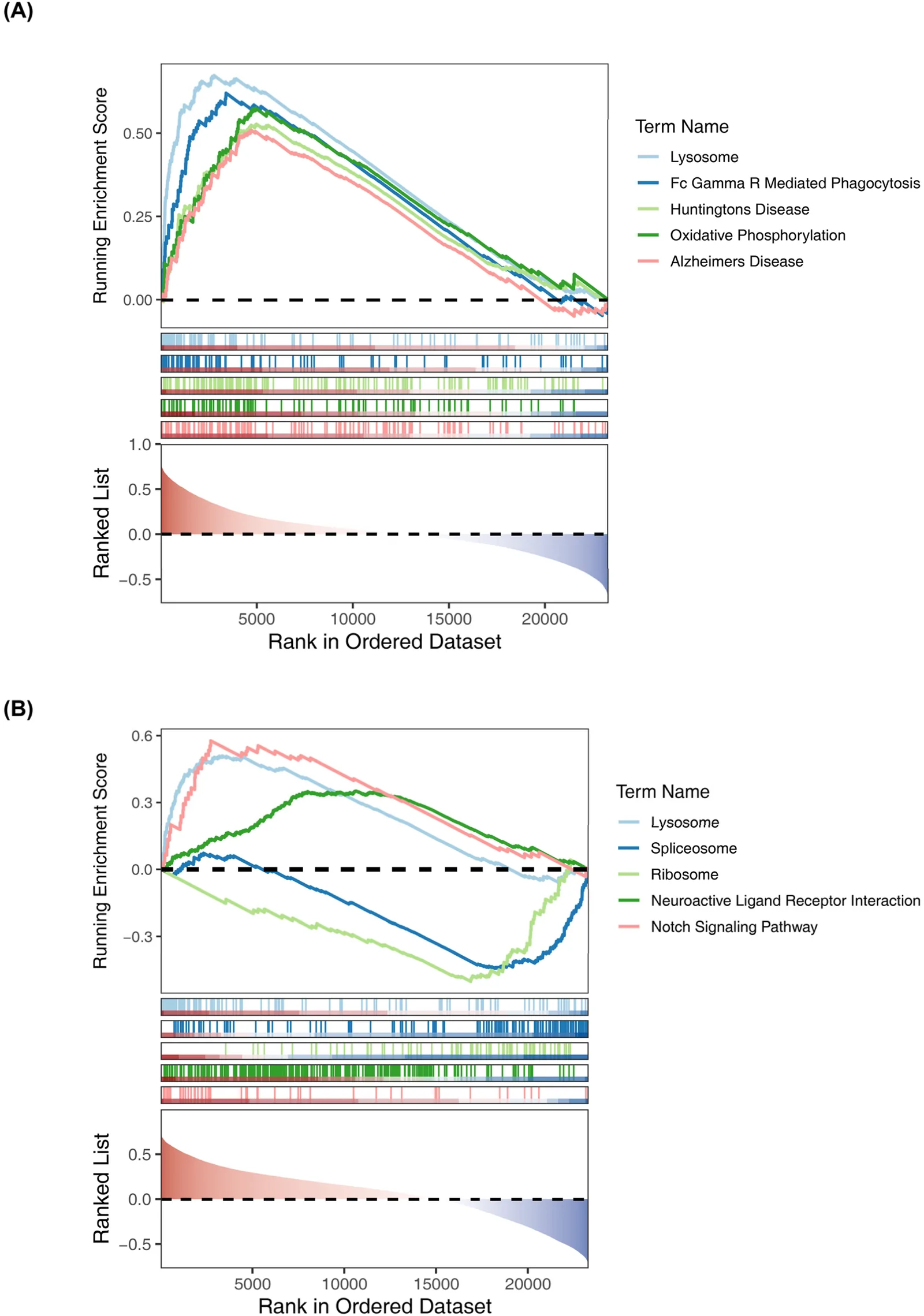
Gene Set Enrichment Analysis (GSEA) of key genes. **(A)** GRB2 **(B)** MAPK3.

We utilized a correlation analysis package to investigate the relationship between these two key genes and found that MAPK3 and GRB2 exhibited a significant positive correlation (cor = 0.4367, *p* < 0.05). (Figure 7). To further reveal the potential function of the 6 diagnostic biomarkers, GSEA was performed based on each single hub gene (Figures 8A,B). By performing GSEA analysis of each key genes, we found that GRB2, MAPK3 were related to “FC GAMMA R mediated phagocytosis”.

### Prediction of potential TF/miRNA-diagnostic biomarkers regulatory network

**FIGURE 9:**
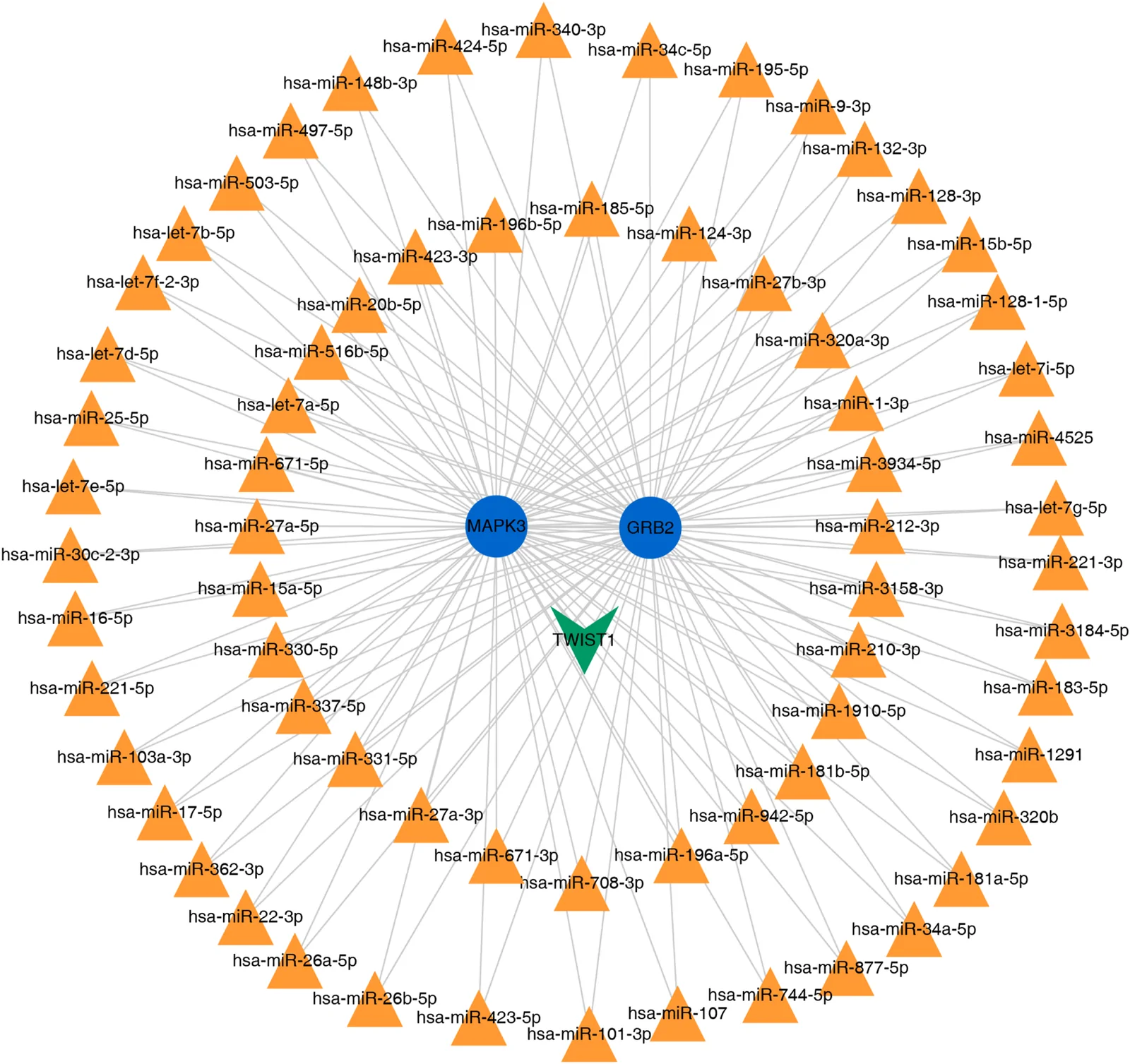
miRNet-predicted regulatory network of 2 diagnostic key genes.

The miRNA and TFs regulatory network of the 2 key genes was predicted using miRNet. We choose the miRNA of degree >2 for visualization. As illustrated in (Figure 9), the interaction network comprised a total of 71 nodes and yielded 137 edges.

### Validation of key genes in myocardial infarction PBMCs

**FIGURE 10:**
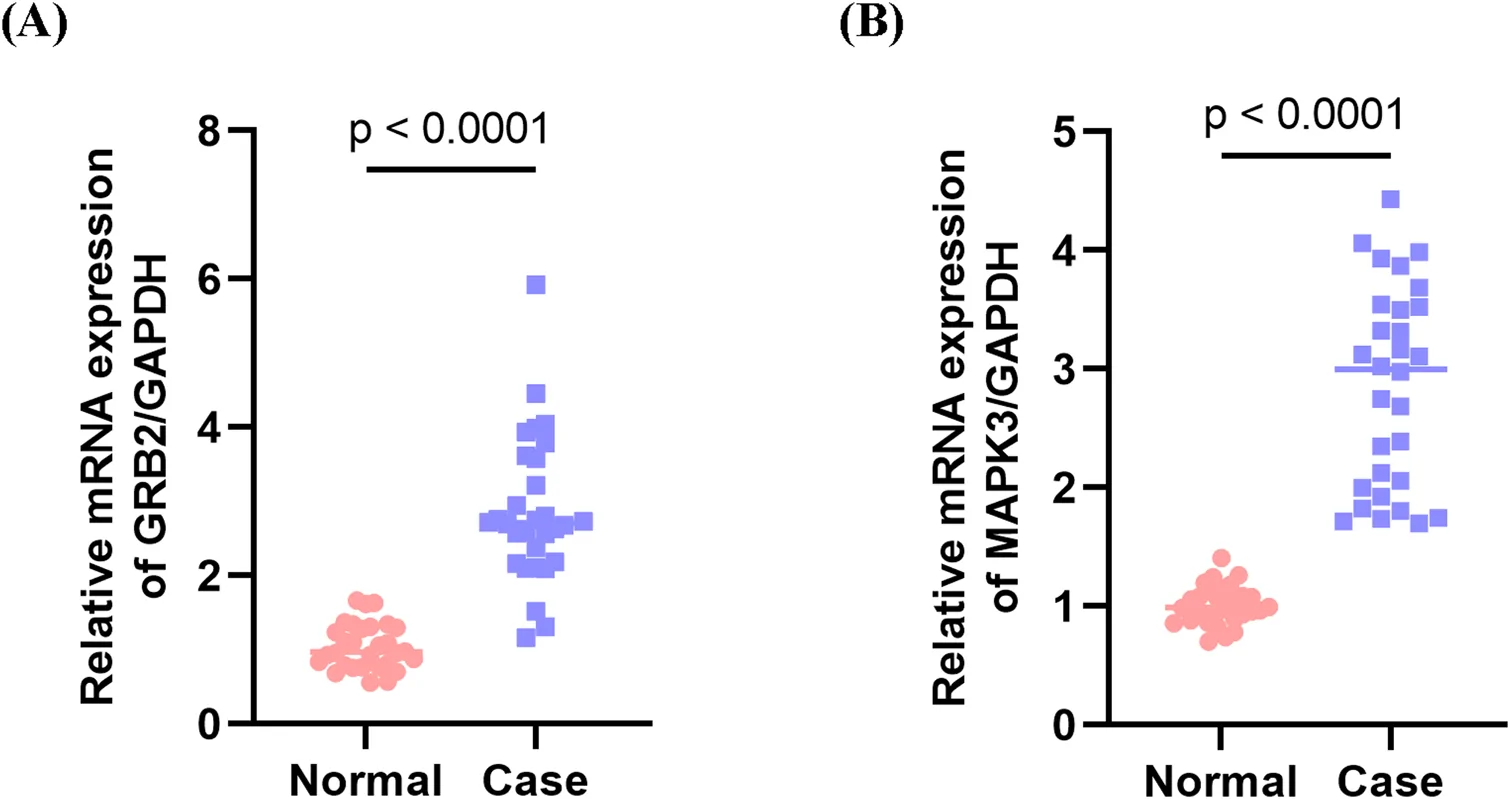
Validation of key genes in peripheral blood mononuclear cells (PBMCs) from myocardial infarction (MI) patients. **(A)** Expression levels of MAPK3 in PBMC samples from MI patients and non-MI controls. **(B)** Expression levels of GRB2 in PBMC samples from MI patients and non-MI controls. Both MAPK3 and GRB2 were significantly upregulated in MI patients compared with controls, consistent with the bioinformatics predictions.

Quantitative PCR validation conducted on PBMC samples from MI patients revealed significant upregulation of two key genes compared to non-MI controls. Specifically, GRB2 (*p* < 0.0001) and MAPK3 (*p* < 0.0001) exhibited marked increases, which was consistent with the bioinformatics findings (Figures 10A,B).

### Validation of candidate genes in murine myocardial infarction model

**FIGURE 11:**
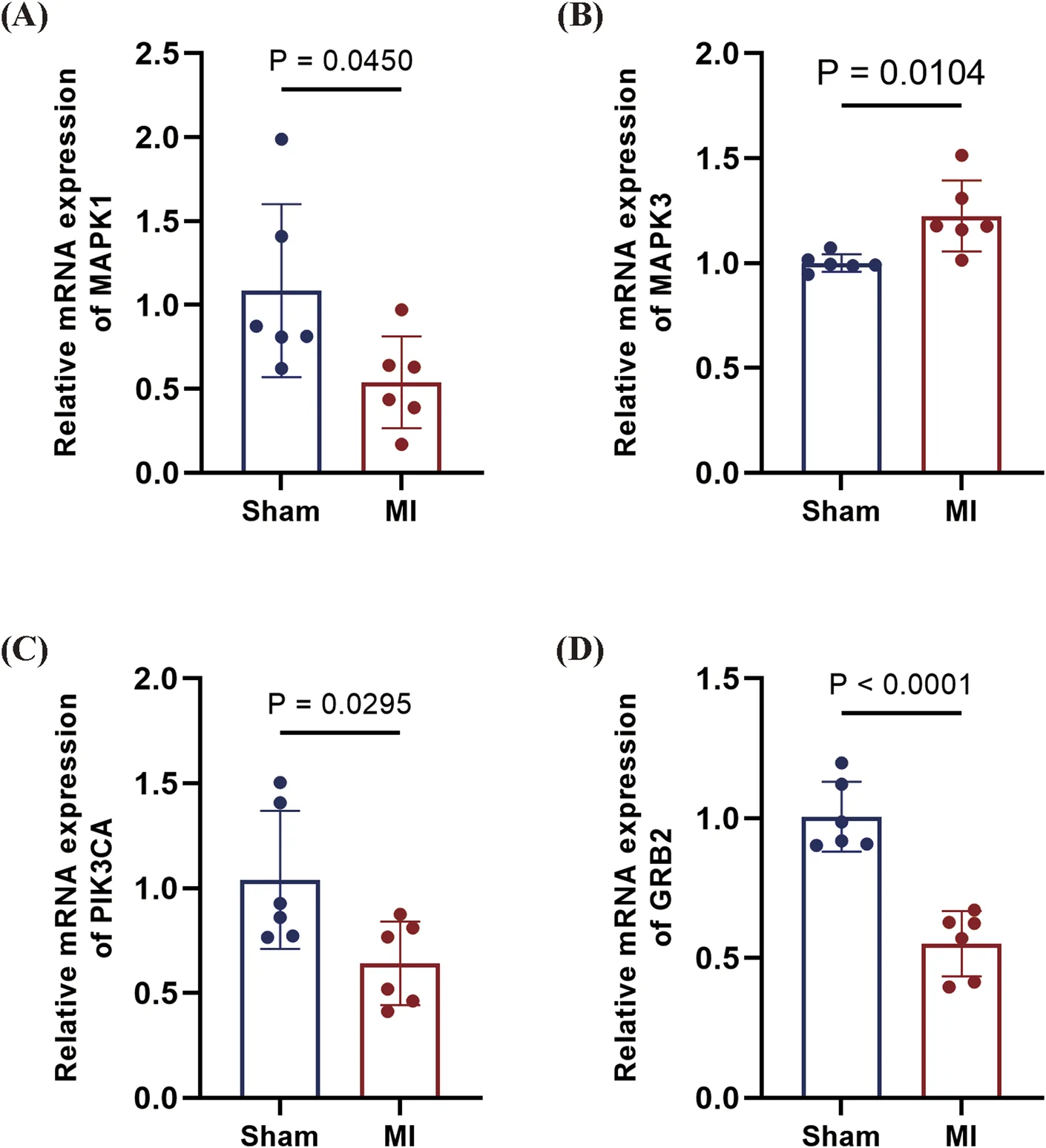
qPCR validation of candidate genes in mouse MI model. **(A)** MAPK1 downregulated in MI myocardium (*P* = 0.0450). **(B)**, MAPK3 upregulated in MI tissue (*P* = 0.0104). **(C)** PIK3CA downward trend in MI (*P* = 0.0295). **(D)** GRB2 profoundly downregulated in MI (*P* < 0.0001). Data are presented as mean ± SD. Statistical significance was assessed by Student’s t-test (n = 6 per group).

To corroborate clinical findings, we examined mRNA expression of GRB2, MAPK3 (ERK1), and PIK3CA in a murine myocardial infarction model (genes selected based on AUC >0.6 in clinical cohort). qPCR analysis revealed the following results: significant downregulation of MAPK1 in infarcted myocardium; marked upregulation of MAPK3; and downward trends for PIK3CA and GRB2. The expression patterns of MAPK1, MAPK3, and PIK3CA aligned with those observed in human peripheral blood profiles. Notably, GRB2 exhibited discordant regulation, being downregulated in murine cardiac tissue while upregulated in the PBMCs of STEMI patients (AUC = 0.85). This divergence suggests tissue-specific and/or stage-dependent regulatory mechanisms in infarction pathogenesis (Figures 11A–D).

## Discussion

Acute myocardial infarction (AMI) is characterized by abrupt thrombotic coronary occlusion, typically resulting from rupture or erosion of atherosclerotic plaques. This process exposes plaque-derived thrombogenic components that trigger platelet activation, adhesion, and aggregation, concurrently activating the coagulation cascade to form an occlusive intraluminal thrombus (Falk, 1985). Platelets serve as primary regulators of hemostasis yet paradoxically drive myocardial infarction (MI) pathogenesis through dual thrombotic and inflammatory mechanisms: atherosclerotic plaque-induced thrombosis and amplified vascular inflammation (Corti et al., 2003; Gawaz, 2006).

This study identified two differentially expressed genes (DEGs) in the platelet transcriptome that can effectively distinguish STEMI patients. ROC curve analysis demonstrated that these two genes exhibit high sensitivity and specificity for STEMI, suggesting their potential as biomarkers for diagnosing myocardial infarction (MI). Validation using clinical samples and animal models further confirmed the clinical relevance and diagnostic value of these genes. Mitogen-activated protein kinase 3 (MAPK3), although widely recognized for its role in tumor progression (Taherkhani et al., 2023),as recently been shown to play a crucial role in myocardial injury. Inhibition of MAPK3 significantly reduces ischemia-induced cardiomyocyte apoptosis (Liu et al., 2018), and MAPK3 has been identified as a central target in platelet activation signaling (Fan et al., 2024). Excessive platelet activation is considered a key step in thrombus formation during acute myocardial infarction (AMI) (Hally et al., 2017). Additionally, the adaptor protein GRB2 has been shown to mediate JAK2 tyrosine phosphorylation, contributing to fibrosis formation in the heart (Chen et al., 2022), Negative regulation of MiR-378 inhibits collagen deposition and reduces the degree of fibrosis (LncRNA PCFL promotes cardiac fibrosis). Wu et al. (Wu et al., 2018) also confirmed a negative correlation between GRB2 expression and recovery of cardiac function after MI. Furthermore, GRB2 plays a crucial role in platelet activation signaling (Dütting et al., 2014). Our study revealed that MAPK3 and GRB2 are significantly co-enriched in the “platelet activation” pathway, suggesting a potential interaction between them in STEMI (ST-segment elevation myocardial infarction). We hypothesize that ischemic stimuli may regulate the expression of MAPK3 and GRB2, thereby activating platelet activation signaling and contributing to the onset of MI. Therefore, the MAPK3-GRB2 axis not only holds promise as a dual molecular biomarker for early diagnosis of STEMI but may also serve as a potential therapeutic target, with the potential to inhibit thrombosis and alleviate adverse myocardial remodeling. However, this hypothesis requires further investigation and validation.

We observed an interesting paradox: histological analysis and peripheral blood clinical validation indicated that GRB2 was upregulated in MI patients (AUC = 0.85); However, qPCR results from the MI mouse model showed a significant downregulation of GRB2 in local myocardial tissue. This discrepancy may be due to multiple factors, including small sample size and sample heterogeneity. Clinical samples were primarily derived from peripheral blood mononuclear cells of acute-phase patients, whereas animal experiments focused on chronic infarct and surrounding fibrotic tissues. Such heterogeneity could lead to biased results. Moreover, the gene expression patterns of peripheral blood mononuclear cells differ from those of platelets or cardiomyocytes. GRB2 may exhibit cell-specific expression fluctuations during platelet activation, but the mixed cell population in peripheral blood could obscure these changes, leading to discrepancies. This suggests the need for larger sample sizes and the potential use of single-cell sequencing technologies to analyze the expression of target genes in specific functional cells, thereby mitigating the effects of cellular heterogeneity.

Transcriptomic profiling of platelet function-related genes reveals quantitative alterations preceding acute myocardial infarction. Converging evidence from our data and existing literature implicates GRB2, MAPK3 (ERK1) as critical mediators of cardiovascular risk and thrombogenesis in both murine models and MI patients. Collectively, this study identifies novel infarction-associated diagnostic markers whose dual utility as therapeutic targets and diagnostic biomarkers warrants functional validation and clinical translation to unlock their potential in precision cardiology.

## Strengths and limitations

This study systematically integrates bioinformatics approaches, including GEO dataset analysis, GO/KEGG enrichment, and PPI network construction, with experimental validation using human peripheral blood and a mouse model of acute myocardial infarction (AMI), demonstrating methodological rigor. Two potential diagnostic biomarkers, GRB2 and MAPK3, were identified, both showing promising diagnostic performance (AUC > 0.7), underscoring their clinical translatability. However, several limitations should be noted: First, the relatively small sample size in the clinical validation phase may limit statistical power, potentially reducing the generalizability of the findings. Second, significant discrepancies in the expression of key genes such as GRB2 between human peripheral blood and mouse myocardial tissue were observed. This spatiotemporal heterogeneity, as well as its association with sample type, time points of detection, and underlying regulatory mechanisms, remains unclear and warrants further investigation. Moreover, as clinical samples were obtained from peripheral blood, they may not fully reflect platelet function, which could affect the interpretation of the results. Additionally, the mouse model only assessed gene expression 14 days post-surgery, thus failing to capture the dynamic changes that occur during the acute infarction phase. This limitation may have led to the omission of crucial early regulatory events, hindering a comprehensive understanding of the gene’s role at various stages of infarction. Finally, the specific functions of the identified genes have not yet been validated through cellular experiments, and the mechanistic insights are still at a preliminary stage. To address these limitations, future research should focus on several key areas: expanding the sample size by incorporating multi-center studies with diverse ethnic groups and age stratification to further validate the diagnostic efficacy of the candidate genes; establishing a time-series sample repository from a STEMI mouse model and collecting patient samples from different stages of disease progression to explore the spatiotemporal dynamics of gene expression; obtaining platelet samples and related datasets for further research; utilizing *in vitro* models to investigate the specific mechanisms of the candidate genes; and developing a multi-marker diagnostic model in conjunction with traditional biomarkers to enhance the accuracy of early STEMI diagnosis, thus providing a more robust foundation for clinical translation.

## Conclusion

This study identified GRB2 and MAPK3 as two platelet function-related genes that may serve as potential diagnostic biomarkers for STEMI. Enrichment analysis indicated their involvement in hemostasis and platelet activation pathways. ROC curve analysis confirmed their high diagnostic accuracy (AUC > 0.7). Validation using human PBMCs and a mouse model further supported the clinical relevance and diagnostic value of GRB2 and ERK1 (MAPK3), suggesting their potential role in risk stratification and precision diagnosis of STEMI.

## Data Availability

The original contributions presented in the study are included in the article/Supplementary Material, further inquiries can be directed to the corresponding author.
