# Correction: Identification of platelet function-related genes in STEMI patients

**DOI:** 10.3389/fgene.2026.1992823

**Published:** 2026-10-13

**Authors:** Jingjing Zhu, Shuangya Yang, Qing Guo, Yifan Yang, Bei Shi

**Affiliations:** Department of Cardiology, Affiliated Hospital of Zunyi Medical University, Zunyi, China

**Keywords:** STEMI, key gene, platelet-related genes, diagnosis, CVD

In the published article, there was an error in Figure 1 as published. During the final submission process, Figure 1 was inadvertently replaced with an incorrect image. The corrected Figure 1 and its original caption appear below.

**FIGURE 1:**
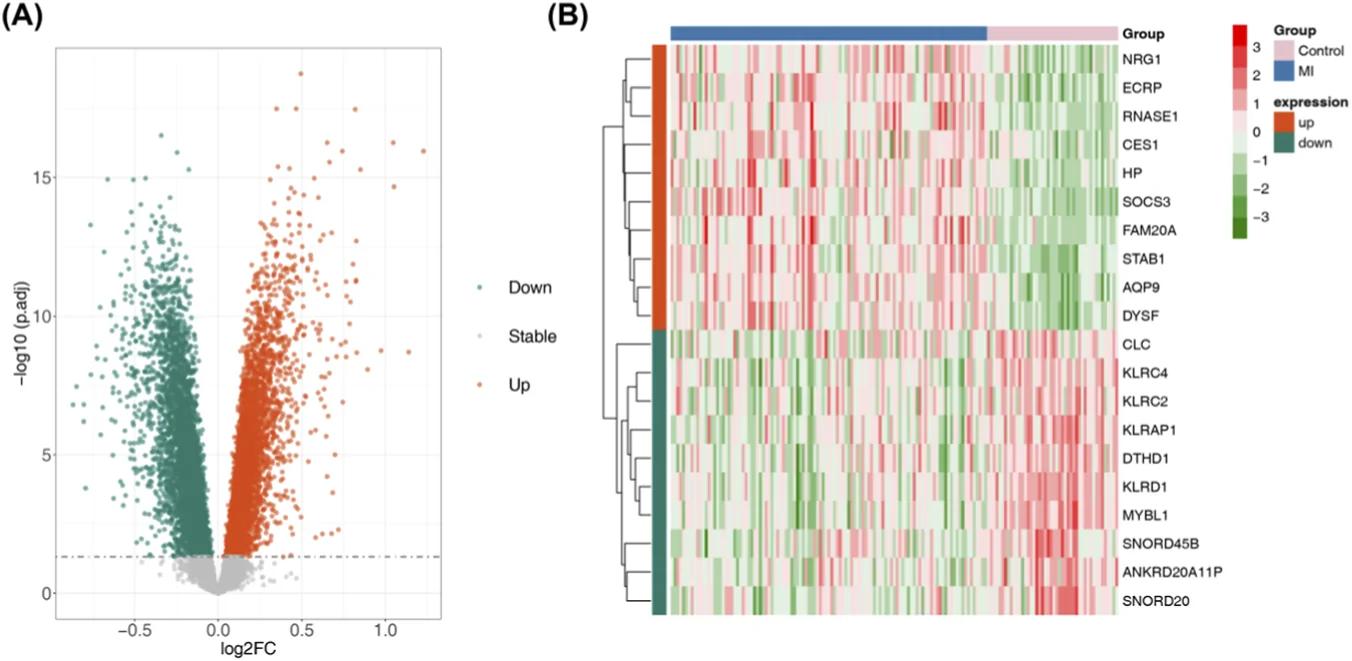
**(A)** Volcano plot of DEGs between MI and control samples in GSE59867. 12,617 DEGs identified (4,457 upregulated and 8,160 downregulated; MI vs. control). **(B)** Heatmap of DEG expression profiles in GSE59867 (MI vs. Control).

The original version of this article has been updated.

