# Supplementary material for: Identification of platelet function-related genes in STEMI patients
Source: Front Genet. 2025 Sep 22;16:1651794. doi: 10.3389/fgene.2025.1651794 (PMC12497627; doi:10.3389/fgene.2025.1651794)
Supplement: Supplementary file 1 [file Table1.docx]

Table S1.Primer sequences for genes in mouse and human.

| Gene | Primer sequences |
| --- | --- |
| PIK3CA (mouse) | Forward: 5'-CCA CGA CCA TCT TCG GGT G-3'  Reverse: 5'-ACG GAG GCA TTC TAA AGT CAC TA-3' |
| GRB2 (mouse) | Forward: 5'-TCC GAG AGA GCG AGA GC-3'  Reverse: 5'-ACT TCA CCA CCC ACA GGA-3' |
| MAPK1 (mouse) | Forward: 5'-GCC TTA CTC TAC TTC TCC CCA-3'  Reverse: 5'-CTG CCT CTG ACT TCT GAA TGC-3' |
| MAPK3(mouse) | Forward: 5'-TCC GCC ATG AGA ATG TTA TAG GC-3'  Reverse: 5'-GGT GGT GTT GAT AAG CAG ATT GG-3' |
| β-actin (mouse) | Forward: 5'-GTG CTA TGT TGC TCT AGA CTT CG-3'  Reverse: 5'-ATG CCA CAG GAT TCC ATA CC-3' |
| PIK3CA (human) | Forward: 5'-GGACCCGATGCGGTTAGAG-3'  Reverse: 5'-ATCAAGTGGATGCCCCACAG-3' |
| PIK3R1 (human) | Forward: 5'-TGTAGTGGTGGACGGCGAAGTA-3'  Reverse: 5'-CATTGAGGGAGTCGTTGTGCTG-3' |
| GRB2 (human) | Forward: 5'-GAGCCAAGGCAGAAGAAATGC-3'  Reverse: 5'-CACCTGTTCTATGTCCCGCA-3' |
| AKT1 (human) | Forward: 5'-GCACAAACGAGGGGAGTACA-3'  Reverse: 5'-AAGGTGCGTTCGATGACAGT-3' |
| MAPK3 (human) | Forward: 5'-ACATCCACTCCGCCAACG-3'  Reverse: 5'-TCAGCCAGAATGCAGCCC-3' |
| MAPK1 (human) | Forward: 5'-TCAGTGAGCGAGACATCCTG-3'  Reverse: 5'-TCCATCACGAAGATGCGGTC-3' |
| GAPDH (human) | Forward: 5'-CGAAGGTGGAGTCAACGGATTT-3'  Reverse: 5'-ATGGGTGGAATCATATTGGAAC-3' |
